# Extrapleural Approach for Thoracic Vertebral Stabilization

**DOI:** 10.1093/icvts/ivag161

**Published:** 2026-05-26

**Authors:** Igor E Konstantinov, Vincent H Le, Michael B Johnson, Amine Mazine

**Affiliations:** Department of Cardiothoracic Surgery, Royal Children’s Hospital, Melbourne, Victoria, 3052, Australia; Department of Paediatrics, University of Melbourne, Melbourne, Victoria, 3052, Australia; Heart Research Group, Murdoch Children’s Research Institute, Melbourne, Victoria, 3052, Australia; Melbourne Centre for Cardiovascular Genomics and Regenerative Medicine, Melbourne, Victoria, 3052, Australia; Department of Cardiothoracic Surgery, Royal Children’s Hospital, Melbourne, Victoria, 3052, Australia; Department of Orthopaedics, Royal Children’s Hospital, Melbourne, Victoria, 3052, Australia; Department of Cardiothoracic Surgery, Royal Children’s Hospital, Melbourne, Victoria, 3052, Australia

**Keywords:** extrapleural approach, thoracic vertebral stabilization, traumatic injury

## Abstract

Surgical access to the upper thoracic vertebral bodies, defined as T1-T5, remains a technical challenge. The anterior extrapleural approach provides excellent visualization and access to the thoracic vertebrae; however, it remains a challenge due to the presence of the great vessels, sternum and trachea. Herein, we describe the case of a 16-year-old boy with avoidant/restrictive food intake disorder and autism spectrum disorder, who presented with lower limb paraparesis following a traumatic hyperflexion injury. Imaging revealed a T2 pathological burst fracture due to spinal haemangioma and osteopenia. Thoracic vertebral decompression and stabilization were performed through an anterior extrapleural approach using a median sternotomy combined with a lateral cervical incision. This approach provided excellent exposure of the anterior upper thoracic vertebral bodies.

## INTRODUCTION

Access to the upper thoracic vertebral bodies, defined as T1-T5, remains a surgical challenge. Although several approaches have been described, many provide limited access to the upper thoracic vertebrae.^1–4^ The anterior extrapleural approach provides excellent visualization and wide operative access to the cervicothoracic junction and upper thoracic vertebrae for surgical intervention.

Herein, we detail a technique for the extrapleural approach to thoracic vertebral stabilization. The Royal Children’s Hospital Human Research Ethics Committee (HREC/21/QCHQ/80891) approved this retrospective review on November 11, 2021, and the parents of the patient provided informed written consent for the publication of the data.

## CASE REPORT

**Figure 1.**
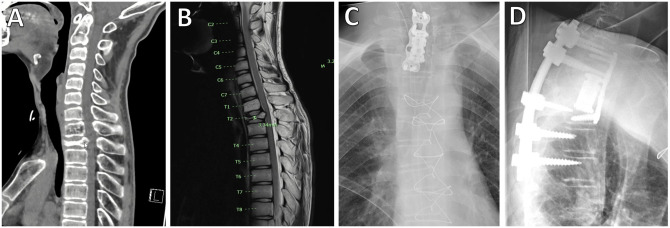
Pre-Operative and Post-Operative Imaging. (A) Pre-operative CT. (B) Pre-operative MRI. (C) Radiographic imaging following anterior plate fixation. (D) Radiographic imaging following posterior plate fixation.

A 16-year-old male adolescent (height: 182 cm, weight: 60.5 kg) with avoidant/restrictive food intake disorder and autism spectrum disorder presented to hospital with lower limb paraparesis and urinary incontinence following a traumatic spinal hyperflexion injury. Computed tomography and magnetic resonance imaging demonstrated a T2 pathological burst fracture due to haemangioma and osteopenia with spinal cord compression (**Figure 1**). He underwent thoracic vertebral decompression and stabilization through an anterior extrapleural approach.

**Figure 2.**
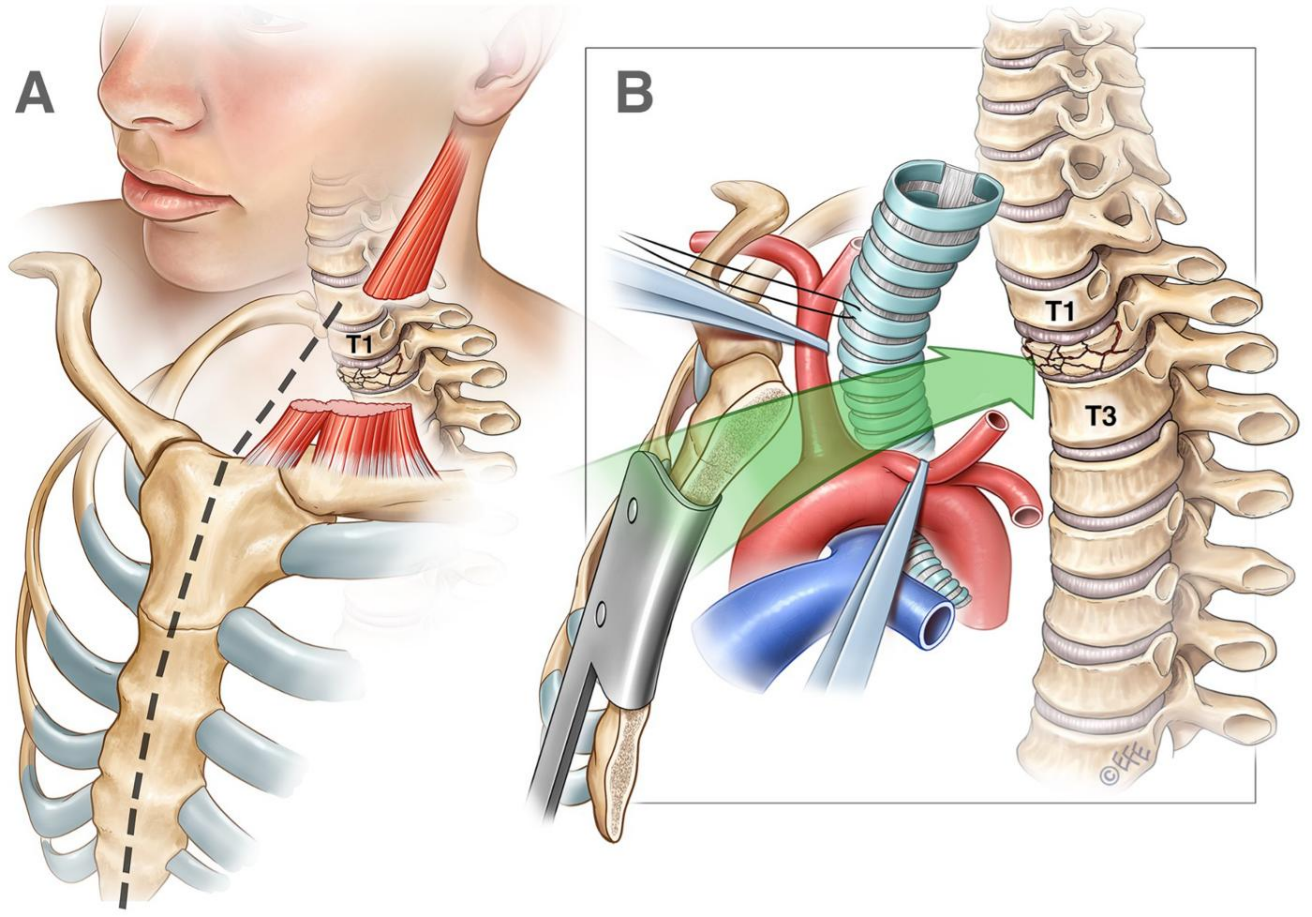
Illustration of the Surgical Approach. (A) The line of incision to approach the anterior thoracic vertebra. No muscles were divided. Part of the sternocleidomastoid muscle is not drawn by the artist to illustrate the location of the T1 vertebral body (B), sternotomy and mobilization of the great vessels and trachea.

A left-sided cervical incision and median sternotomy were performed to facilitate anterior extrapleural access. Deep dissection was carried out in an anterior-to-posterior plane to expose C7-T4 vertebral bodies. The brachiocephalic artery and left common carotid artery were gently retracted inferiorly using vascular loops, and the trachea was retracted medially (**Figure 2**, **Video 1**). Care was taken to mobilize these structures gently and under direct vision to minimize traction-related injury and maintain a safe operative corridor. An anterior cervical retractor was positioned, allowing for exposure of the cervico-thoracic spine. T2 Corpectomy of the compressed T2 vertebral body was performed, followed by T1-T3 anterior interbody fusion and C7-T3 anterior plate fixation. Due to osteopenia, the sternum was approximated using a figure-of-eight interlocking sternal wires technique.^5^ On postoperative day 4, the patient underwent a planned second-stage operation for posterior spinal stabilization (**Figure 1**). At the 1-year follow-up, the patient was well. He was able to walk short distances unassisted and had normal bladder and bowel function. The fixation was stable on physical exam and radiography. He continues to undergo outpatient physical rehabilitation for long-term recovery.

## COMMENT

Anterior approach surgery to the cervical and upper thoracic vertebrae is uncommon and is reserved for traumatic spinal injury requiring extensive anterior vertebral decompression and stabilization.^1–4^ The anterior approach to the thoracic spine via a median sternotomy was first described by Cauchoix and Binet in 1957.^1^ However, there remained limitations associated with this approach, particularly the requirement to divide the sternohyoid and sternothyroid muscles resulting in higher postoperative morbidity and the limited exposure to vertebral bodies superior to T1, such as C7.^2^ Due to higher rate of sternal wound infection in the early era of cardiothoracic surgery, the notion was to avoid sternotomy at all costs as these patients often had associated osteopenia. In patients with complex thoracic vertebral pathology, an extrapleural approach, via a median sternotomy and lateral cervical incision, may be used to facilitate extensive anterior decompression, stabilization and reconstruction of the vertebrae. In the case reported herein, the patient presented with a T2 burst fracture and spinal cord compression which required a broad operative field to perform direct anterior decompression, T2 corpectomy, interbody fusion, and anterior plate fixation.

The surgical technique described herein provided wide exposure of the cervicothoracic junction and upper thoracic vertebral bodies without division of the cervical strap muscles. It also allowed controlled mobilization of the brachiocephalic artery, left common carotid artery, and trachea under direct vision. Care was taken during exposure to minimize traction-related injury to these structures, and development of the extrapleural plane avoided pleural entry and additional intrathoracic morbidity. Importantly, to reduce sternotomy-related postoperative complications, we used figure-8 interlocking sternal wires technique for effective sternal stabilization. This is particularly important in patients with osteopenia or poor bone quality.^4^

Several alternative approaches have been described, including manubriotomy, thoracotomy, thoracoscopic, and posterior-only approaches (**Table S1**). Each offers distinct advantages and limitations, depending on the level of pathology and the operative objectives. For example, a more limited exposure such as manubriotomy may provide adequate access to the C6–T3 vertebrae. In contrast, although a posterior-only approach avoids mediastinal dissection, it may not provide sufficient access for the treatment of anterior vertebral pathology.^1–4^

In conclusion, thoracic vertebral decompression and stabilization can be performed with excellent exposure through an extrapleural approach, via a midline sternotomy and lateral cervical incision. We believe this technique is feasible and reproducible and that it should be performed more often in complex upper thoracic vertebral trauma requiring anterior stabilization.

## Supplementary Material

ivag161_Supplementary_Data

## Data Availability

The data underlying this article are available in the article and in its online supplementary material.

## References

[1] Cauchoix J, Binet JP. Anterior surgical approaches to the spine. Ann R Coll Surg Engl. 1957;21:234-243. 13470743

[2] Fuentes S, Malikov S, Blondel B, Métellus P, Dufour H, Grisoli F. Cervicosternotomy as an anterior approach to the upper thoracic and cervicothoracic spinal junction. J Neurosurg Spine. 2010;12:160-164. 20121350 10.3171/2009.9.SPINE09471

[3] Islam S, Hresko MT, Fishman SJ. Extrapleural thoracoscopic anterior spinal fusion: a modified video-assisted thoracoscopic surgery approach to the pediatric spine. JSLS. 2001;5:187-189. 11394435 PMC3015433

[4] Fessler RG, Dietze DD Jr, Millan MM, Peace D. Lateral parascapular extrapleural approach to the upper thoracic spine. J Neurosurg. 1991;75:349-355. 1869932 10.3171/jns.1991.75.3.0349

[5] Konstantinov IE, Saxena P. Sternal stabilization by interlocking wires: an alternative simple technique for high-risk patients. J Card Surg. 2009;24:510-511. 19538225 10.1111/j.1540-8191.2008.00800.x

