# Supplementary material for: Extrapleural Approach for Thoracic Vertebral Stabilization
Source: Interdiscip Cardiovasc Thorac Surg. 2026 May 26;41(7):ivag161. doi: 10.1093/icvts/ivag161 (PMC13457916; doi:10.1093/icvts/ivag161)
Supplement: Supplementary file 1 [file ivag161_Supplementary_Data.docx]

**Supplementary Table 1.** Description of various approaches to the cervicothoracic vertebrae.

| **Approach** | **Typical exposure** | **Strengths** | **Limitations** |
| --- | --- | --- | --- |
| Manubriotomy / cervicothoracic | Approx. C6 to T3 | Less extensive than full sternotomy, useful for upper cervicothoracic junction | Limited and narrower exposure |
| Sternotomy with cervical extension | Approx. C7 to T4/T5 | Wide anterior exposure, direct access for decompression and stabilisation | Full sternotomy, requires mobilisation of great vessels and trachea |
| Thoracotomy | Mid to lower thoracic vertebrae | Familiar exposure, wide lateral access | Limited access to upper cervicothoracic junction |
| Thoracoscopic | Selected thoracic levels | Less invasive, reduced chest wall trauma | Technically demanding, limited working space, less suitable for complex reconstruction |
| Posterior-only | Posterior elements, indirect anterior access | Avoids anterior dissection | Cannot provide access for extensive anterior decompression or vertebral body reconstruction |
